# Hysteroscopic removal of intrauterine device in early pregnancy

**DOI:** 10.1186/s12905-022-02012-5

**Published:** 2022-10-27

**Authors:** Guglielmo Stabile, Caterina Godina, Francesco Cracco, Francesco Paolo Mangino, Melania Canton, Federico Romano, Giuseppe Ricci

**Affiliations:** 1 Department of Obstetrics and Gynaecology, Institute for Maternal and Child Health-IRCCS “Burlo Garofolo”, 34137 Trieste, Italy; 2 Department of Medicine, Surgery and Health Sciences, University of Trieste, 34100 Trieste, Italy

**Keywords:** IUD, Intrauterine device, Hysteroscopy, Embryoscopy, Ultrasound, Pregnancy, Contraception

## Abstract

**Background:**

Intrauterine devices (IUDs) are commonly used as contraceptives worldwide. However, pregnancies in patients carrying this kind of device may occur. IUD removal when the woman wishes to continue their pregnancy may be very challenging. Only 9 manuscripts in literature reported such similar procedure.

**Case presentation:**

We report the case of an hysteroscopic removal of IUD in a young woman at 6 weeks of gestation.

**Discussion:**

The case reported highlights safety and efficacy of operative hysteroscopy as a method of IUD removal in early pregnancy, although other different methods have been reported in literature. In our opinion, maintaining a low infusion pressure during the procedure may help avoiding potential gestational sac damage and IUD displacement for better grasping.

**Supplementary Information:**

The online version contains supplementary material available at 10.1186/s12905-022-02012-5.

## Background

Intrauterine devices (IUDs) are considered both safe and highly effective contraceptives. In the first year after insertion, failure rates for copper IUD (Cu-IUD) and levonorgestrel-releasing IUD are approximately 0.8 and 0.2%, respectively [1]. Nevertheless, pregnancies may rarely occur with an IUD in situ.

It is now well established that pregnancies that occur with an IUD in situ are at increased risk for complications such as ectopic pregnancies [2, 3], miscarriages, preterm delivery, and chorioamnionitis. Additional concerns exist regarding the potential teratogenic risk to the fetus owing to levonorgestrel-releasing IUD exposures, although there is no direct evidence to support this claim [4].

Regarding methods to retrieve IUDs in pregnancy where the strings are not clinically accessible, ultrasound and hysteroscopic methods have been used to guide IUD retrieval when strings were non visible, with good success rates. Both methods have been associated with 35 to 45% perinatal loss rates due to miscarriage, extremely preterm birth or infection, but these compare favorably with 50% perinatal loss in series where pregnancy continued with a retained IUD [5].

We report a case of a woman with a copper Intrauterine device (IUD) for contraception at 6 weeks of gestation. Transvaginal ultrasound confirmed the presence of a fetal pole with cardiac activity and showed two fragments of IUD dislocated near the right Fallopian tubal ostium. After thorough counseling with the patient about the risks, considering patient’s desire to preserve the pregnancy, we decided to perform an hysteroscopic removal of the fragments.

## Case presentation

A 37-year-old nulliparous woman was sent by her physician after an intrauterine pregnancy was confirmed despite the presence of a copper intrauterine device (IUD) for contraception, which had been inserted 3 years before (Euorgine™, Ancora 375 Cu). Since the strings were easily visible, her attending physician attempted to remove the IUD using ring forceps, but the arms of the device remained in the cavity. The woman complained an intermittent pelvic pain lasting about 2 weeks and was admitted to the gynecological clinic of an Italian tertiary care hospital. The physical examination showed uterus slightly larger than normal, mobile, not very painful when mobilized. IUD threads were no more visible at the external os of the cervix as the central portion of the IUD had already been removed. A transvaginal ultrasound scan showed an intrauterine pregnancy at 6 weeks of gestation with fetal pole with cardiac activity and two fragments of IUD dislocated near the right fallopian tubal ostium.

After adequate counseling with the patient, hysteroscopic removal of the IUD was performed. Transvaginal preoperative ultrasound using a Voluson S10 (General Electric Healthcare GE, Zipf, Austria) equipped with endovaginal and transabdominal probes confirmed the previous ultrasound finding.

Hysteroscopy was conducted by a gynecologist with extensive gynecological hysteroscopic expertise using a Bettocchi 5 mm Hysteroscope (Karl Storz Endoskope). The exam was carried with the patient in a lithotomic position with an empty bladder, under sedation and without cervical dilatation.

**Fig. 1:**
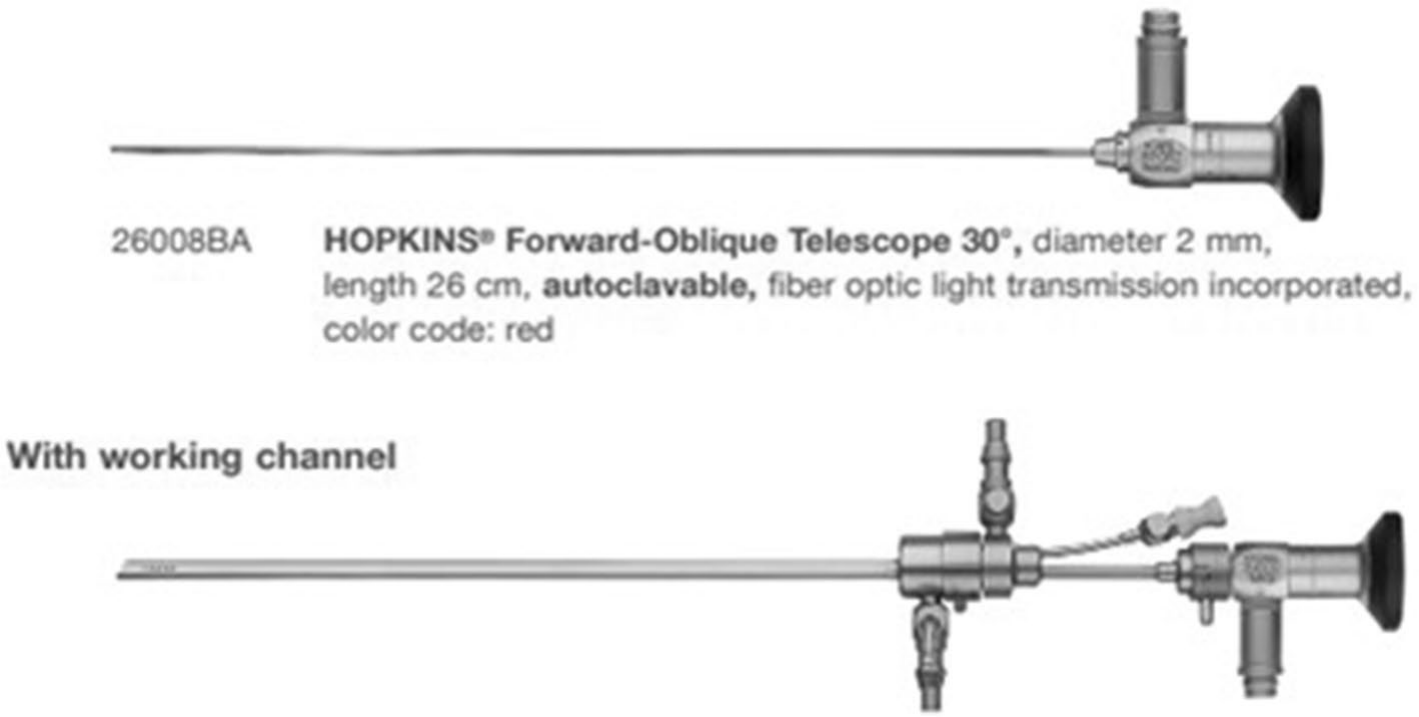
Bettocchi hysteroscope, 5 mm (Karl Storz). Working channel allows the use of semi-rigid 5 Fr. operating instruments

**Fig. 2:**
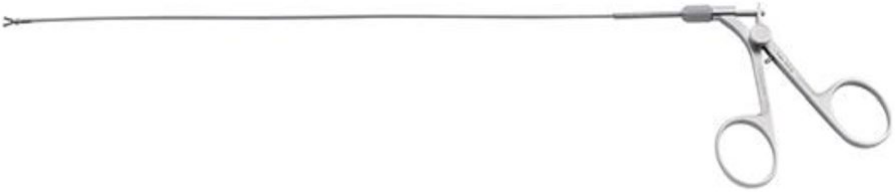
Semi-rigid and Reusable 5 Fr. Operating Instrument: Biopsy Forceps. (Karl Storz)

An operative fiber optic hysteroscope (5 mm in outer diameter) with biopsy forceps was used. The distending medium, 0.9% normal saline, was infused at low pressure (intrauterine fluid pressure was initially maintained at 80 mmHg during the access in the uterine cavity, then reduced to 40 mmHg) from a bottle suspended approximately 60 cm above the uterus and connected to the hysteroscopic irrigation channel by an intravenous infusion set. The hysteroscope was introduced very gently into the cervical canal under direct visualization and advanced into the uterine cavity to find the fragments. The two fragments were individuated and very carefully removed directly one by one with a small forceps. The procedure was performed on an outpatient basis and the procedure was successful on the first attempt, maintaining low infusion pressure after the individuation of the two arms of the device, without uterine bleeding after the procedure (Video). The instruments are pictured in (Figs. 1 and 2).

**Fig. 3:**
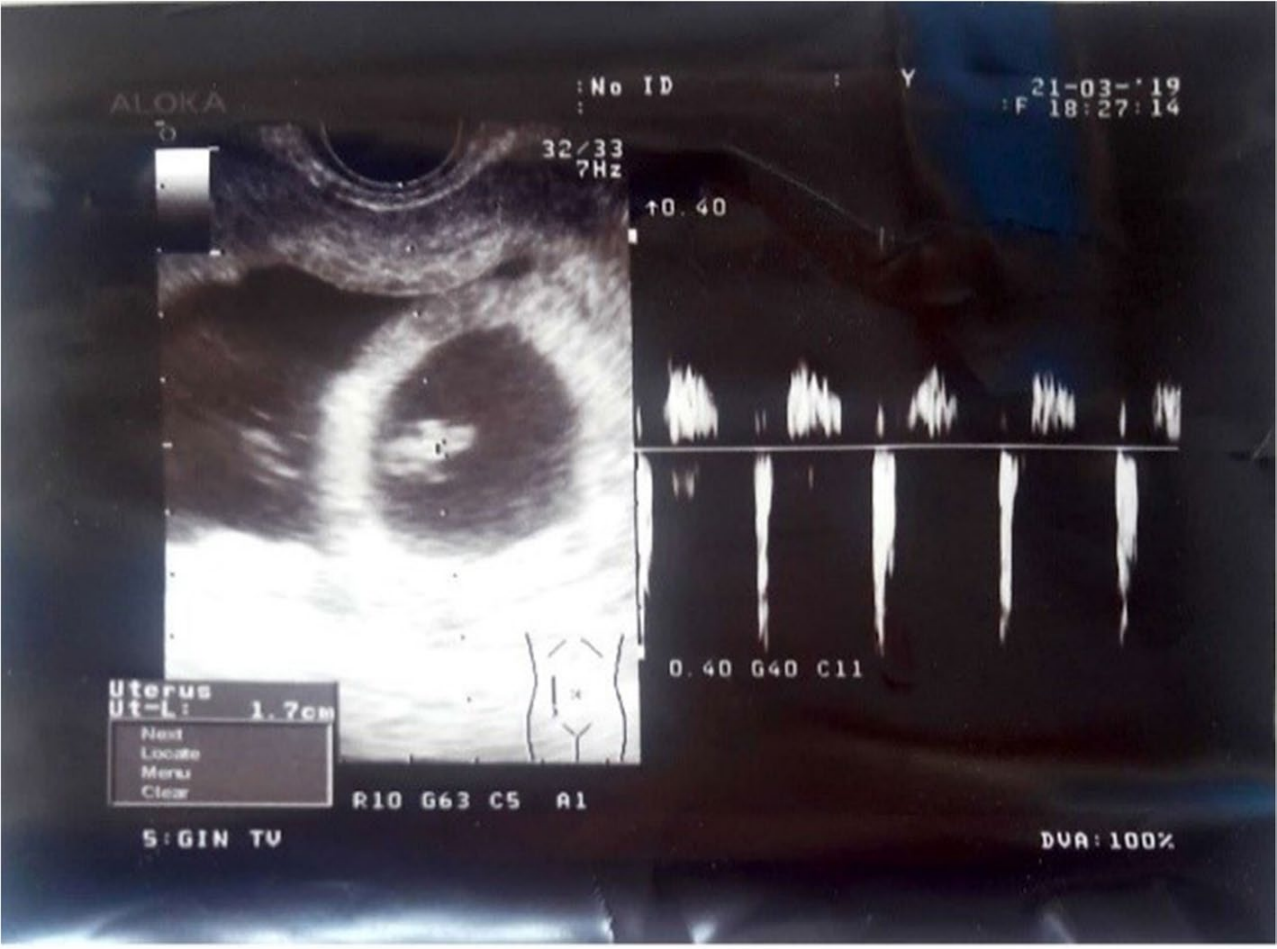
Transvaginal ultrasound scan after the procedure

**Fig. 4:**
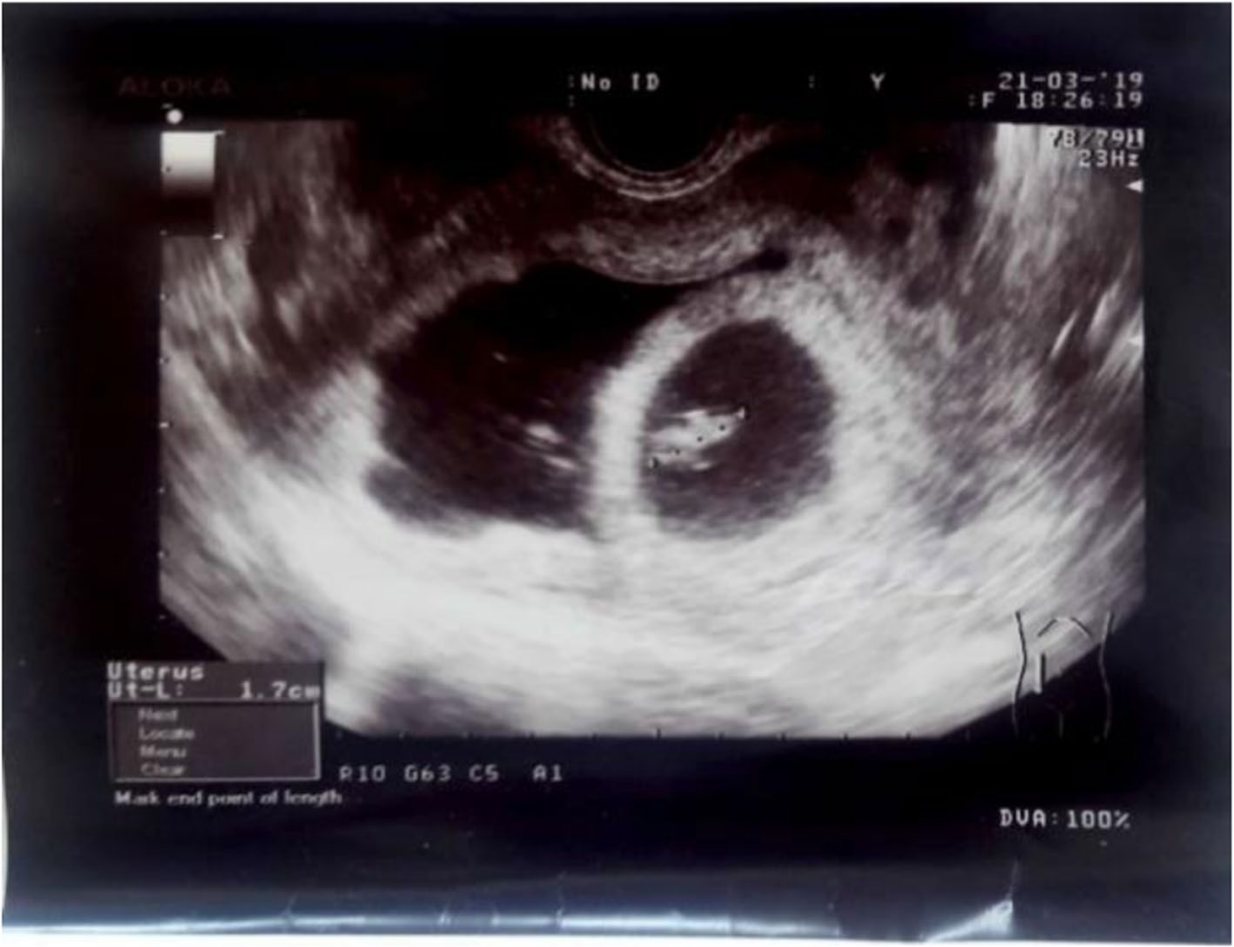
Transvaginal ultrasound scan after the procedure

At the end of the procedure, a transvaginal ultrasound scan revealed an intact gestational sac with a viable embryo (Figs. 3 and 4).

Follow-up ultrasonography to check the gestational sac was performed also at 2 days and 1 week after removal of the IUD’s fragments. No antibiotic therapy was administered after the procedure.

One month later, the pregnancy was regularly ongoing with single viable embryo. However, due to the counseling that informed the patient about the low risk of miscarriage and fetal damage, the patient decided to interrupt the pregnancy. The termination of pregnancy was performed medically according to our voluntary termination of pregnancy protocol (administration of Mifepristone 600 mg and 48 h later Misoprostol 400 micrograms, the latter of which can be repeated after 8 hours) [6]. The termination of pregnancy occurred regularly and the subsequent follow up was negative, with negative blood beta human chorionic gonadotropin after 30 days.

## Discussion and conclusions

Removal of IUDs in early pregnancy is a delicate and difficult procedure, especially when the patient wishes to continue her pregnancy and/or IUD threads are not visible at the external os of the cervix. Some authors suggested [7, 8] that when IUD threads are not visible, the IUD should be left inside the uterine cavity, in order to avoid a potential abortion following the removal. Nevertheless, severe complications may occur when the IUD is left inside the uterine cavity with an ongoing pregnancy, which may affect both the mother and the fetus. Mermet et al. [9] followed 67 women with copper IUD that wished to continue their pregnancies. Thirty-eight of them had the IUD removed, while in 29 of them the IUD were kept in place. A significantly increased risk of adverse outcome was reported in the latter group: miscarriage risk rate was increased in women with IUD kept in place when compared with women who had their IUD removed (48% vs 8%). Moreover, preterm delivery risk and premature rupture of membranes (PROM) risk were increased in pregnancy with retained IUDs (90% vs 34%). Other adverse outcomes reported were vaginal bleeding, intrauterine infections, and fetal congenital anomalies.

In summary, the choice regarding removing the IUD rather than leaving it in place is tough and must be shared together with the woman and her partner, making them part of the decision process. In addition to that, IUD removal procedure may be challenging for the physician.

Three main techniques have been reported in literature: hysteroscopic, ultrasound-guided hysteroscopic, or ultrasound-guided forceps IUD removal. Due to the rarity of the condition, no single technique has been deemed superior. Likely, substantial differences in the availability of equipment and expertise in different centers may limit the generalizability of certain techniques [10].

In 2018 Sanders et al. [11] presented a video which demonstrated a surgical approach for a successful removal of IUDs in early pregnancy. Technical tips included using a small-caliber hysteroscope and infusion of small volumes of isotonic distension media. Hysteroscopic IUD removal was successfully performed in all four cases presented. After the procedure, all four patients delivered live births at term. In our experience, a further reduction of the distension medium flow was useful to efficiently grasp the fragments and to reduce the likelihood to damage to the gestational sac, since fragments mobility were reduced and their removal was therefore easily accomplished. Given the delicacy of the procedure, and the patient’s emotional involvement which may alter her pain perception, hysteroscopy performed in an outpatient setting might not be the procedure of choice [12].

The hysteroscopic procedure should be of course performed very carefully and cervical dilation should be avoided, thus maximum 5 mm hysteroscopes must be used. During the procedure, a low-pressure infusion should be maintained in order to lessen the damage to the gestational sac and to easily grasp IUD fragments. Since the procedure carries a non-negligible risk of iatrogenic abortion, it must be performed only by operators with high experience in hysteroscopy. According to our review, a thorough counseling must be performed, informing patients of a 10% risk of abortion, and preterm delivery rate of about 12%.

## Supplementary Information

**Additional file 1: Video.** IUD removal video. Hysteroscopic removal of intrauterine device in early pregnancy.

## Data Availability

All data generated or analyzed during this study are included in this published article and its supplementary information files.

## Abbreviations

IUD: Intrauterine device
PROM: Premature rupture of membranes
